# Special Issue “Current Research on Omics of Microorganisms”

**DOI:** 10.3390/ijms26178553

**Published:** 2025-09-03

**Authors:** Rosa María Martínez-Espinosa

**Affiliations:** 1 Multidisciplinary Institute for Environmental Studies “Ramón Margalef”, University of Alicante, Ap. 99, E-03080 Alicante, Spain; rosa.martinez@ua.es; Tel.: +34-965903400 (ext. 1258 or 8841); 2 Biochemistry and Molecular Biology and Edaphology and Agricultural Chemistry Department, Faculty of Sciences, University of Alicante, Ap. 99, E-03080 Alicante, Spain

The term “omic” is derived from the Latin suffix “ome”, meaning “mass” or “many”. Thus, “omics”-based studies involve a significant number of measurements per endpoint, rather than one or a few. In connection with research in Life Sciences, “omics” studies integrate the measurement of many parameters related to genes, proteins, lipids and, in general, any biomolecule that can be identified as a target for massively analysing or monitoring specific processes of interest [1]. 

It is difficult to identify the exact date of the beginning of “omics” technologies/disciplines in Life Science research. Some researchers state that the first omics technology, genomics, appeared in the literature of the 1980s, and that the term (supposedly coined by Tom Roderick in a bar in 1986) is the combination of two words: *gene* and *chromosome* [2,3]. However, it is difficult to establish the exact moment that genomics began and was consolidated as a new discipline of science. 

Probably, the completion of the Human Genome Project [4], alongside reference genomes for several other organisms (most of them microorganisms), catapulted genomics into being a powerful tool for transforming science, mainly in areas closely related to biology and medicine [5]. In parallel, advances in technological facilities, not only for the massive analysis of gene sequences, but also for the analysis of all biomolecules in general, have decreased the cost of gene sequencing and biomolecular analysis, leading to the practice becoming a staple in research [6]. Shortly before genomics’ consolidation as a discipline, three other omics quickly appeared: transcriptomics, proteomics, and metabolomics (Figure 1).

Currently, different “omics”-based disciplines can be distinguished (Table 1), making multi-omics integration that allows for the monitoring of static genomic alterations, temporal transcriptomic perturbations, and alternative splicing possible, as well as spatial–temporal proteomic dynamics and post-translational modifications [7].

In this context, the construction of knowledge regarding biological systems implies the analysis of comprehensive causal relationships between molecular signatures and phenotypic manifestations because of a specific single-cell response to changes in environmental parameters or due to a particular disease, thus offering additional resolving power that enables investigations at a single-cell level in a wide spectra of research areas, including the search for new biotechnological applications (medicine and ecology biogeochemical cycles, etc.) [6,8,9].

Finally, and in connection with microbiomics, metagenomics (also known as “microbial environmental genomics”) is revealed as a powerful tool for studying the genetic material of communities of microorganisms present in a specific environment, without the need for prior cultivation (which constitutes a significant advancement in the characterisation of microbial biodiversity, mainly in those extreme environments that make the isolation, characterisation, and in vitro growth of the extremophilic microbial strains difficult) [10,11,12]. The impact of metagenomics has been so far-reaching in the advancement of knowledge that, over the last decade and a half, a technological revolution has taken place, leading to the development of next-generation metagenomic sequencing [13,14,15].

This profusion of disciplines, together with technological advancement, has made it possible for science at the forefront of knowledge in the field of Life Sciences to advance by leaps and bounds in the last three decades, enabling the discovery of thousands of articles published in indexed international scientific journals, as well as patents. As part of the generation of new knowledge and the exploitation of research results, this Special Issue (in which five original articles are integrated) aims to contribute to the advancement of knowledge on omics to better understand microbial metabolism and physiology, microbial biodiversity, potential applications of microorganisms and their molecules, and the relationships between microbial communities inhabiting a specific ecosystem. Below, each of these works is briefly described in chronological order of publication and grouped by broad areas of study for which “omics” disciplines have been used.

The first article [16] is a good example of how the traditional taxonomic classification of microorganisms could benefit from the analysis of 16S rRNA sequences obtained by metabarcoding using new algorithms: in this case, the DADA2 algorithm. The fine-tuning of DADA2 parameters for a multiregional metabarcoding analysis of 16S rRNA genes from activated sludge and the subsequent comparison of taxonomy classification power and taxonomy databases revealed that a more accurate classification of OTUs into taxa and higher numbers of OTUs for each region can be achieved. In addition, from the database comparisons of each of the regions studied, the highest numbers of taxonomic groups were obtained using the SILVA database. These results suggest that the standardisation of metabarcoding of short amplicons may be possible.

The second [17] and the fourth [18] articles use different bacterial strains as model organisms to explore cyanide biodegradation using genomics [17] and understand elemental sulphur oxidation through genomics and proteomics [18]. In brief, cyanide biodegradation via members of the genus *Pseudomonas* has been analysed through an in silico comparative genomic approach that was useful for the bioprospection of putative cyanotrophic bacteria and the identification of new genes putatively involved in cyanide biodegradation. The results obtained revealed that one of the two key enzymes involved in cyanide biodegradation, nitrilase NitC, is widely distributed among many taxonomic groups, including the genus *Pseudomonas* (in which the *nit1C* gene cluster that codes for the nitrilase NitC is part of the accessory genome of the genus *Pseudomonas*, being located in 49 genomes of 25 species of this genus). The omics-based study also allowed for the identification of the molecular machineries involved in resistance to cyanide and to chemotaxis or signal transduction co-occurring with the process of cyanide biodegradation [17]. In the study by Rudenko et al. [18], genomic and proteomic approaches were used to characterise the molecular machinery that enables elemental sulphur oxidation via the bacterium *Beggiatoa leptomitoformis*, identifying that persulphide dioxygenase plays a key role in this metabolic process. The results obtained indicate that representatives of the genus *Beggiatoa* may utilise elemental sulphur as a key storage substance and activate enzymatic pathways to obtain energy from it, such as the dissimilatory oxidation of thiosulfate involving the branched Sox system, which produces sulphate and contributes to the reaccumulation of intracellular elemental sulphur. Consequently, members of the *Beggiatoa* genus play a relevant role in the global sulphur biogeochemical cycle [18].

The third article [19] uses genomics to analyse the mitochondrial genomes of the basidiomycete fungus *Thelephora ganbajun*. Overall, this comprehensive analysis began with the sequencing and assembling of the complete mitogenomes of 40 samples exhibiting diverse *cox1* heterogeneity patterns from various geographical origins. Additionally, heterogeneous variants in the *nad5* gene, which, like *cox1*, displayed variability across multiple copies, have been identified. These two mitochondrial genes are crucial because they code for proteins involved in electron transfer associated with cellular respiration. The main findings of this work reveal a wide range of heterogeneity, polymorphisms, and structural variation (the presence of heterogeneous genes, introns, and HEGs was found to greatly affect the composition and evolution of mitochondrial genes). The main insights provide a strong basis for further research on the evolution and effects of mitochondrial heterogeneity in eukaryotes. 

In the fifth article [20], a complete pangenome association analysis has been conducted to identify key evolutionary aspects in the *Chlamydiaceae* family (Gram-negative bacteria characterised by a unique biphasic developmental cycle) as well as factors involved in their relationship with their hosts. By definition, a pangenome is the complete set of genes present within a species, encompassing both the genes shared by all members (termed “the core genome”) and those unique to some individuals (known as “the accessory genome”). Up to 101 genomes from this family have been integrated into the hierarchical clustering of the pangenomic matrix, and the delineation of groups with statistically consistent genetic diversity revealed the formation of two major clades within the *Chlamydiaceae* family, corresponding to the genera *Chlamydophila* and *Chlamydia* (there are a total of 289 differentially abundant genes between the clades, with 129 found exclusively in the genus *Chlamydia* and 160 in *Chlamydophila*). By analysing those genes, it can be concluded that the highest diversity of gene elements related to replication, translation, as well as energy metabolism and the metabolism of amino acids and nucleotides, correlates with the number of hosts they infect, which is relevant information for managing zoonotic transmission, among other concerns.

In summary, these works contribute to the advancement of knowledge, using the “omics” disciplines as a powerful tool for the massive analysis of biological information. However, “omics” technologies, while powerful, face limitations in data integration, statistical rigour, and biological interpretation. These challenges include the heterogeneity across different “omics” platforms, the difficulty of integrating data from various sources, and the need for sophisticated computational tools to analyse the vast amounts of data generated. Apart from these technical limitations, ethical considerations around data privacy and access also pose significant hurdles [1,21]. Consequently, further studies are required in the short term to overcome these limitations.

## Figures and Tables

**Figure 1:**
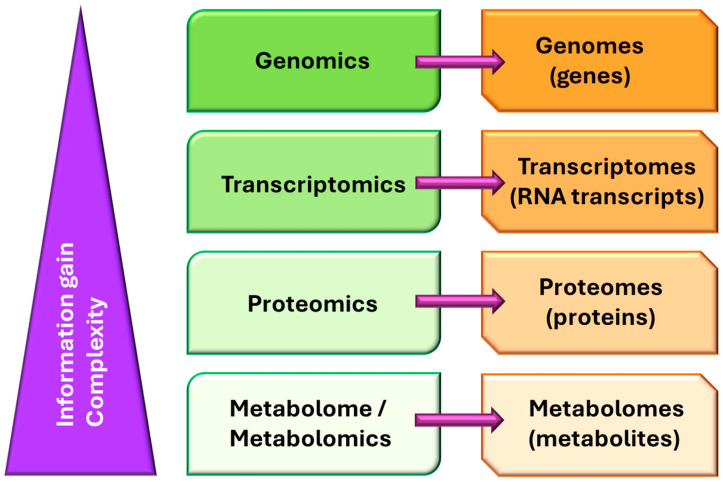
General scheme of the main “omics” disciplines that evolved in the early 1990s, making it possible to use any of the key groups of biomolecules related to the central dogma of molecular biology as targets.

**Table 1:** Current “omic” disciplines.

Name of “Omic” Discipline	Aim
Genomics	Study of genomes
Transcriptomics	Study of RNA transcripts
Proteomics	Study of proteins
Metabolomics	Study of metabolites
Metabonomics	Multiparametric study of metabolic responses of living systems to external stimuli or genetic modifications
Epigenomics	Study of epigenetic modifications
Lipidomics	Study of lipids
Glycomics	Study of glycans and carbohydrates
Microbiomics	Study of microbial communities of an ecosystem
